# Effects of maize rotation on the physicochemical properties and microbial communities of American ginseng cultivated soil

**DOI:** 10.1038/s41598-019-44530-7

**Published:** 2019-06-13

**Authors:** Xiao-Lin Jiao, Xue-Song Zhang, Xiao-Hong Lu, Ruijun Qin, Yan-Meng Bi, Wei-Wei Gao

**Affiliations:** 1 Institute of Medicinal Plant Development, Chinese Academy of Medical Sciences and Peking Union Medical College, Beijing, 100193 China; 2 Oregon State University-Hermiston Agricultural Research and Extension Center, Hermiston, OR 97838 USA; 3 Institute of Plant Protection, Chinese Academy of Agricultural Sciences, Beijing, 100193 China

**Keywords:** Agroecology, Field trials

## Abstract

The production of American ginseng (*Panax quinquefolius* L.) is severely limited by the replant disorders in China. Crop rotation with maize might reduce the replant problems, but little information is available on the effect of maize rotation on soil cultivated with ginseng. In this study, we analyzed nutrients, phenolic acids, and microbial communities in soils from the fields with continuous maize, mono-culture ginseng, and 1-, 3-, and 5-year maize rotation after ginseng. Pot experiments were also conducted to evaluate the performance of replanting ginseng in these soils. The results showed that Mn, Cu, and 5 phenolic acids in ginseng-cultivated soil were significantly decreased by maize rotation. A 5-year maize rotation significantly increased the relative abundance of beneficial soil bacteria, such as *Arthrobacter*, rather than decreasing the abundances of potential pathogenic genera. Clustering analysis revealed that the physicochemical properties and microbial communities of 3- and 5-year maize rotation soil were more similar to CM than to G soil. The biomass of replanted ginseng root was improved, and root disease was reduced over 3 years of maize rotation. Overall, the results showed that at least a 3-year maize rotation is needed to overcome the replant failure of American ginseng.

## Introduction

American ginseng (*Panax quinquefolius* L.) is known for its pharmacological functions, such as anti-oxidation, anti-diabetes, and anti-cancer activities, as well as enhancement of the central nervous system, and it has been consumed in China for more than 300 years^1–4^. Originating in the eastern part of North America^1^, American ginseng was introduced to China in the 1980s. At present, ginseng planting area covers over 10,000 ha in northern China. American ginseng is a perennial plant requiring shade, which usually grows in fields for four years with high inputs (e.g., soil preparation, shading, orchard cleaning, fertilization, disease control) before harvesting. In crop fields, this plant’s growth is hampered by replanting failure, which reduces the root yield dramatically due to replant disease. Consequently, farmers never plant ginseng in the same field due to the crop failure concern; thus, the fields used by farmers for ginseng production have been decreasing dramatically over time. For example, the Huairou District of Beijing was a major ginseng-growing region in China with an area of 700 ha in the 2000s but currently a ginseng planting area less than 2 ha. It has been reported that ginseng replanting failure and diseases have also occurred in North America^5^. It is evident that replant disorders have seriously restricted sustainable American ginseng development.

Replant failure in some crops or fruit trees is attributed to abiotic and biotic factors^6,7^. The abiotic factors generally include imbalanced soil nutrients, improper soil pH, and plant allelopathy or autotoxicity^8–10^. You *et al*. (2015) reported a remarkable decrease in soil pH and exchangeable calcium, ammonium, and total organic carbon with time in soils with *Panax ginseng* growth^8^. He *et al*. (2009) reported that autotoxic phenolic acids in surface soil of fields cultivated with American ginseng inhibited the growth of the next ginseng crop^9^. The biotic factors often refer to the change in microbial communities or the accumulation of soilborne pathogens after years of monoculture^11^. Most fungal pathogens of American ginseng root rot (such as *Fusarium* spp. and *Cylindrocarpon* spp.) can survive in soil for many years^5,12^. Dong *et al*. (2016) reported that in soils cropped with three years of notoginseng, which belongs to the same genus as American ginseng and suffers from replant failure, fungal diversity was significantly decreased, while the relative abundance of pathogens, such as *Fusarium oxysporum*, was significantly increased, which were highly correlated with the notoginseng death rate^13^.

Crop rotation, a traditional effective method for improving soil quality, alleviating replant diseases, and boosting yields, has been reported in variety of crops^14,15^. Studies have shown that crop rotation can increase the nutrient availability and soil organic matter^16–18^. Furthermore, this approach may accelerate the degradation of autotoxic chemicals, thereby weakening autotoxicity^19^. Additionally, rotation with nonhost crops can alter the microbial community structure or break the persistence of pathogens that build up in the monoculture of cucumber and grass, among others^15,20,21^. However, only a portion of microorganisms could be detected in previous studies using culture-dependent or molecular analyses, such DGGE and q-PCR, thereby precluding an understanding of the whole picture of soil microbes^22–24^. Recently, the development of high-throughput sequencing has helped to fully elucidate the soil microorganisms and better understand their relationship with plant disease; however, only a small number of studies to date have been reported on *Rehmannia glutinosa*^25^, *Panax notoginseng*^13^, *Pseudostellaria heterophylla*^26^, vanilla^27^, and cucumber^20^, among others. In a study of replanted *Panax ginseng*, Lee *et al*. (2015) screened out several suitable rotation crops, such as perilla and potato, from 18 plant species, which could reduce root rot disease and promote ginseng growth^28^. However, the mechanism underlying soil improvement by rotation is lacking. To date, data concerning the suitable rotation crop for American ginseng have been lacking, as well as the number of years of planting required to overcome the replant failure of American ginseng.

After American ginseng harvesting, spring maize or wheat is usually planted as the following crop in ginseng-growing regions in China. Maize, in particular, is the major rotational crop cultivated broadly as a food, feed and forage crop. However, it is not clear whether the maize rotation could reduce the replant failure in soils cultivated with American ginseng. To help farmers achieve successful replanting of American ginseng, we conducted this study with the objectives of exploring the effect of maize rotations on the chemical and biological properties of soils in which American ginseng were grown and identifying the appropriate intervals before American ginseng replanting. The details of the study included (1) the evaluation of soil nutrients, phenolic acids, fungal and bacterial communities, and relative abundances of pathogenic fungi in ginseng soils, as affected by maize rotations, and (2) investigation of the growth and occurrence of root disease in replanted American ginseng in soils immediately after ginseng cultivation or after maize rotation in different years. Our results will help to gain a better understanding of the physicochemical characteristics of the rotation crop and the establishment of microbial communities in soil and provide sustainable cultivation solutions for American ginseng-growing regions.

## Results

### Soil nutrients

**Figure 1:**
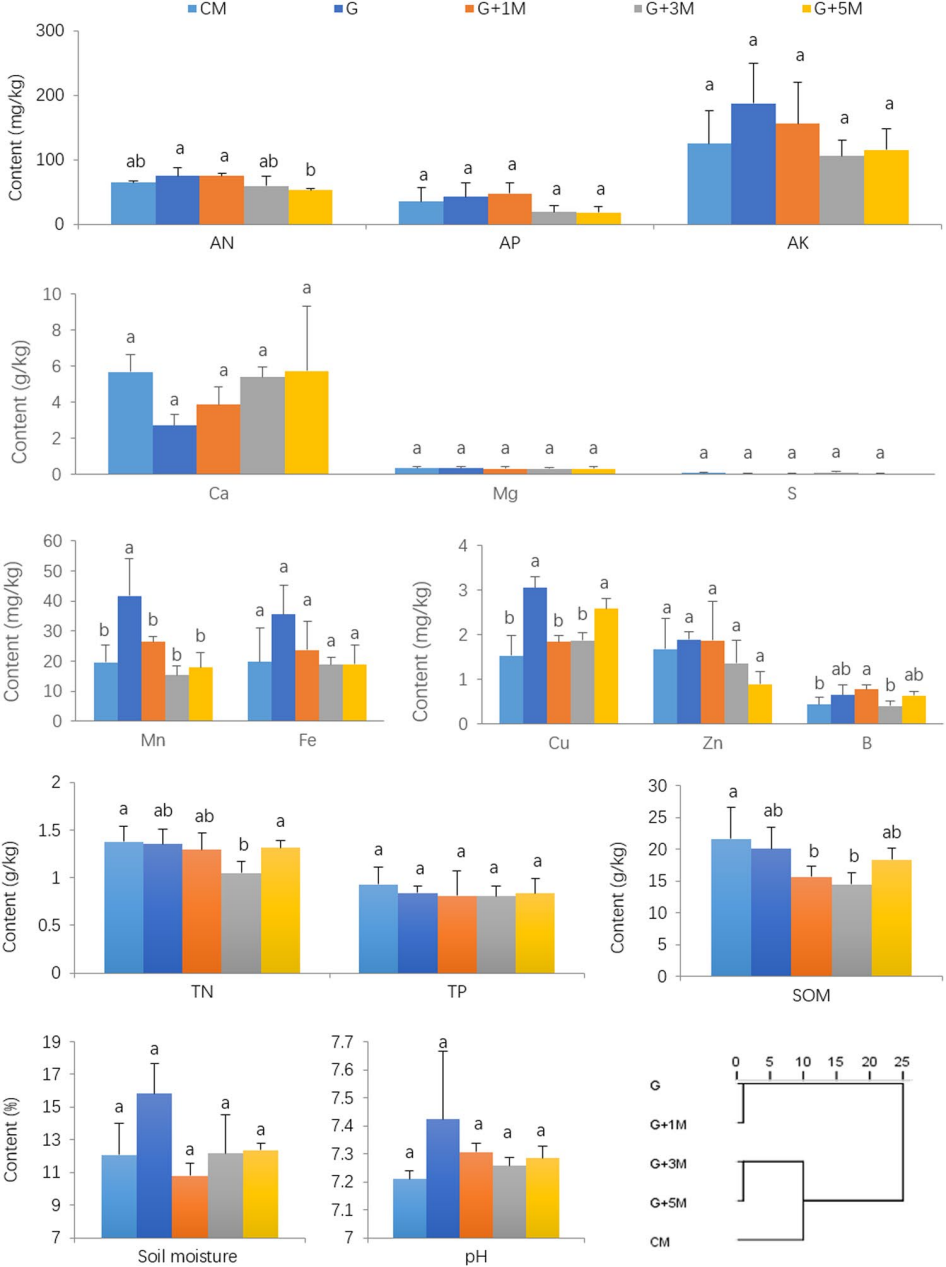
Selected physicochemical properties in soil samples and dendrogram from hierarchical cluster analysis using between-groups linkage method and the squared Euclidean distance. AN = alkali-hydrolysable nitrogen, AP = available phosphorus, AK = available potassium, TN = total nitrogen; TP = total phosphorous, SOM = soil organic matter. Error bars denote the standard deviation of the mean (n = 3) and data designated with the same letter are not significantly different (*P* < 0.05), according to Fisher’s least significant difference method. G = four-year cultivation of American ginseng, M = one-year cultivation of maize, CM = cultivated maize.

Figure 1 shows the data for soil nutrients, SOM and pH in American ginseng-cultivated soil after maize rotation. The concentrations of AN, AP, and AK in American ginseng-cultivated soil (G) tended to decrease with the year of maize rotations, although no significant differences were found. After 3 years of maize rotation, the AN, AP, and AK contents remained similar to the soil of continuously cultivated maize (CM). The available calcium (Ca) tended to increase with the rotation year of maize, while the available magnesium (Mg) or sulfur (S) did not differ among the groups. Micronutrients such as manganese (Mn), copper (Cu), and iron (Fe) were 113%, 99% and 80% higher in G soil than in CM soil, respectively, while they were mostly similar in the soil with the maize crop. No clear pattern was found for Zinc (Zn) and Boron (B) due to the higher variations. The TN and SOM in the G soil tended to decrease after the 3-year maize rotations and then rose to similar levels as in CM soil. No significant differences were observed in any group for TP. The soil pH was approximately 7.2–7.4, and the moisture content was approximately 10.8–15.8% for all soils. Further cluster analysis of the soil nutrient parameters showed that the five soil samples were divided into two groups. One group contained G and G + 1 M, whereas the rest were grouped together (Fig. 1).

### Phenolic acids

**Figure 2:**
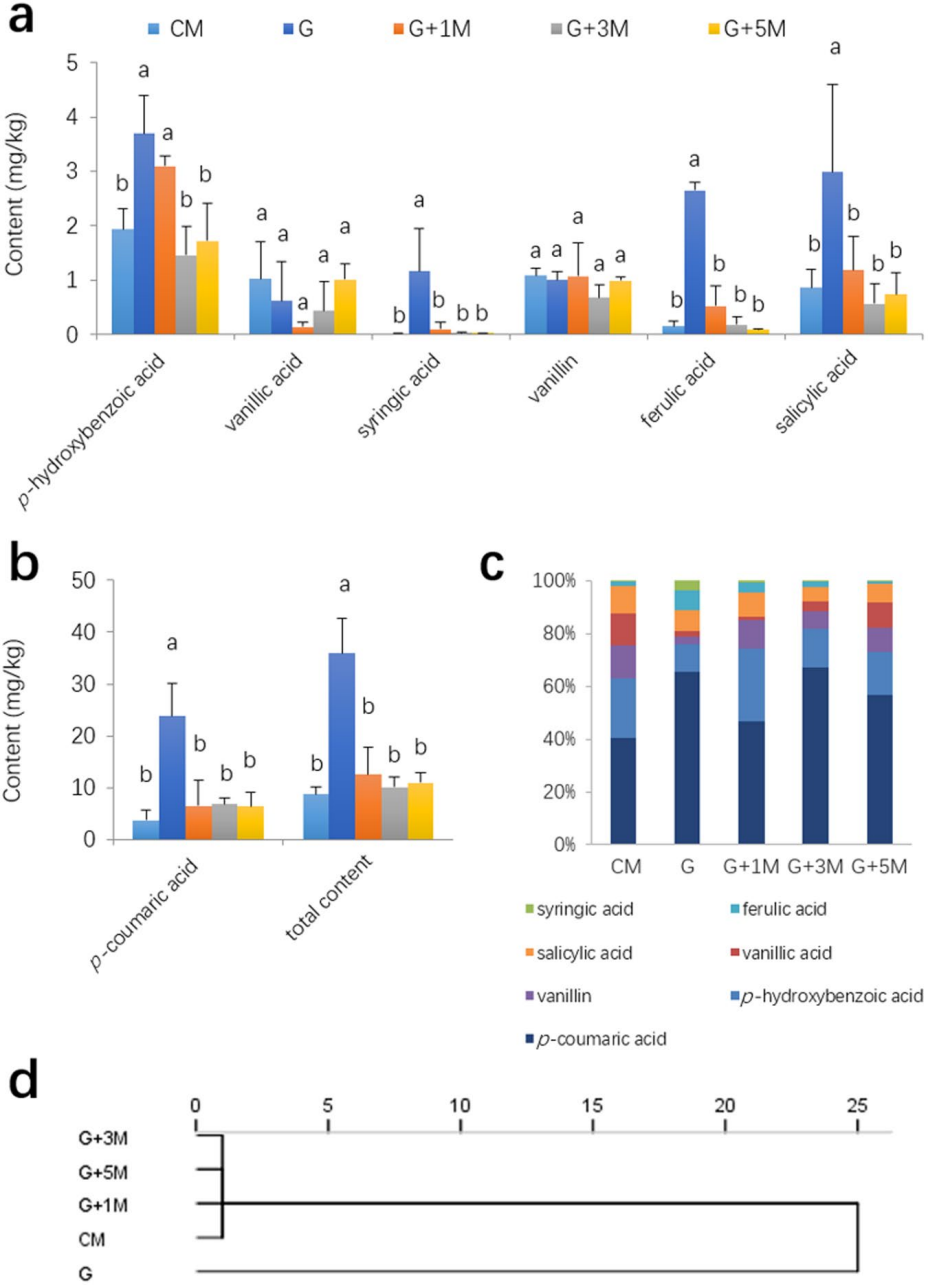
Concentrations (Mean ± SD, n = 3) of the seven phenolic acids (**a**,**b**) and their ratios (**c**) in soil samples and dendrogram from hierarchical cluster analysis using the between-group linkage method and squared Euclidean distance (**d**). Error bars denote the standard deviation of the mean (n = 3), and data designated with the same letter are not significantly (*P* < 0.05) different, according to Fisher’s least significant difference method.

The contents of five phenolic acids in the G soil were ~1 to 48 times those of CM soil (*P* < 0.05), including *p*-hydroxybenzoic, syringic, ferulic, salicylic, and *p*-coumaric acids (Fig. 2). Compared with G soil, the *p*-hydroxybenzoic acid content was significantly decreased after three years of maize rotation, while the other four acid contents were significantly decreased after one year of maize rotation (*P* < 0.05, Fig. 2). However, the contents of vanillic acid and vanillin were similar among all of the groups. The total contents of phenolic acids in the maize planting or rotation groups were significantly less than in G (*P* < 0.05) (Fig. 2b). No obvious change pattern was observed in the ratios of individual phenolic acids during maize rotation (Fig. 2c). Cluster analysis yielded two distinct subgroups; one group involved only G, and the other consisted of four other groups (Fig. 2d).

### Soil fungal and bacterial communities

In all 15 soil samples, 28013–64010 internal transcribed spacer (ITS) final tags were obtained and grouped into 814–1316 fungal operational taxonomic units (OTUs), whereas 37699–58512 16S final tags were obtained and grouped into 2932–3712 bacterial OTUs. The Good’s coverage estimates for all soil samples were >0.970, representing good sequencing depth. The fungal OTUs were assigned into 8 phyla, 20 classes, 60 orders, 109 families, or 264 genera, while the bacterial OTUs were assigned into 40 phyla, 88 classes, 151 orders, 272 families, or 389 genera.

**Figure 3:**
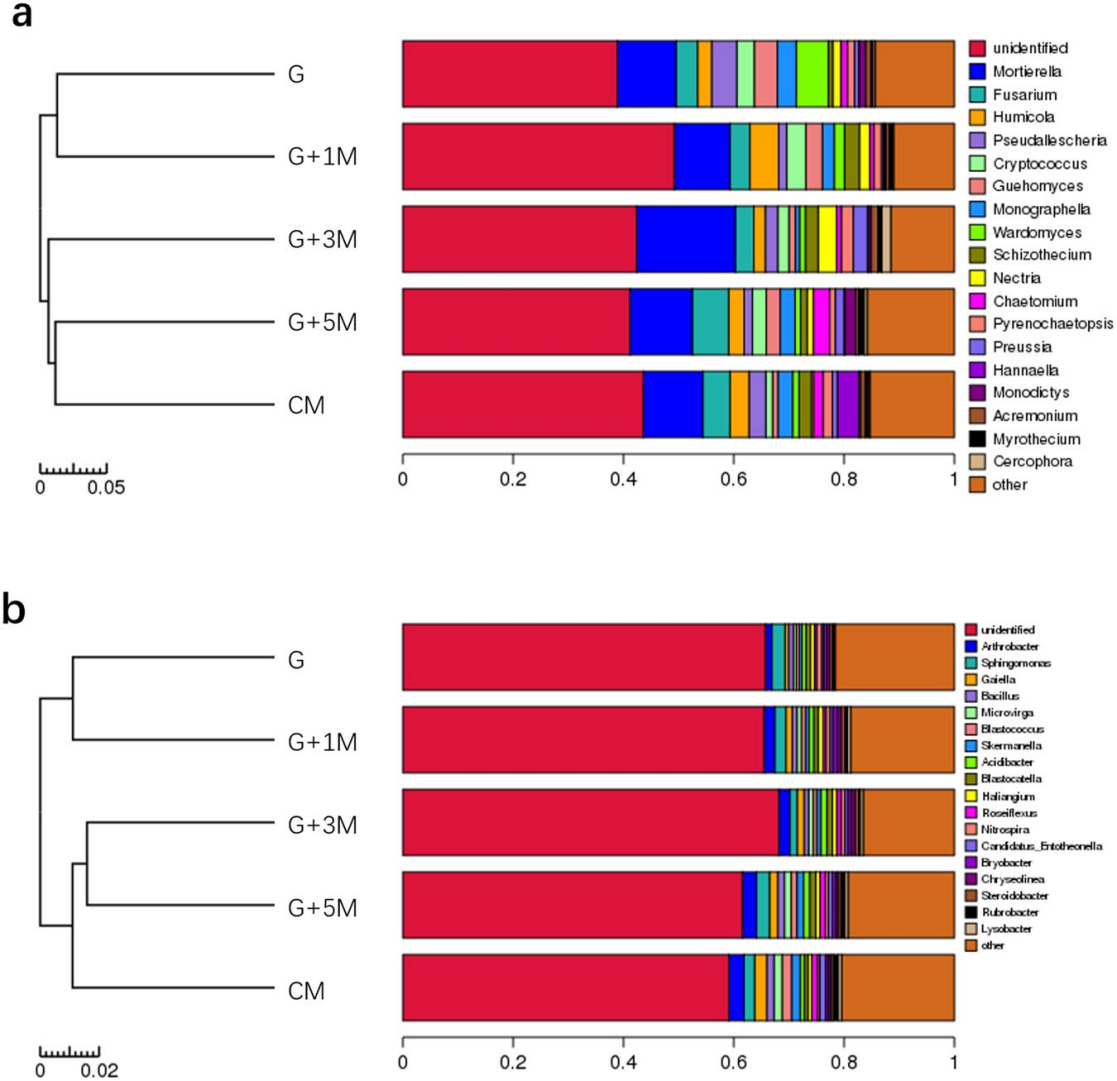
Fungal (**a**) and bacterial (**b**) major genus compositions and hierarchical cluster analysis in the soil samples.

Each genus in all the soil groups was ranked by their average abundance. The sum of the top 20 genera represented 74.56–81.52% of the total identified fungal genera in all samples. The major dominant fungal genera belonged to *Mortierella*, *Fusarium*, *Humicola*, *Pseudallescheria*, *Cryptococcus*, *Guehomyces*, *Monographella*, *Wardomyces*, *Schizothecium* and *Nectria*, among others (Fig. 3). For all the top 20 fungal genera, no significant difference was observed in their relative abundances between G and CM soil (*P* > 0.05). However, *Fusarium* and *Schizothecium* were reduced, while *Guehomyces* and *Wardomyces* were increased, in G compared with CM soil. Additionally, no significant differences were observed among all five soil groups in the relative abundances of fungal functional groups, including potential pathogenic genera, such as *Alternaria* and *Cylindrocarpon*, antagonistic genera, such as *Penicillium* and *Trichoderma*, and mycorrhizae, such as *Glomus* (*P* > 0.05, Table S1).

The sum of the top 20 genera represented 42.85–51.71% of the total identified bacterial genera. The dominant bacterial genera belonged to *Arthrobacter*, *Sphingomonas*, *Gaiella*, *Bacillus*, *Microvirga*, *Blastococcus*, *Skermanella*, *Acidibacter*, *Blastocatella*, and *Haliangium*, among others. Among the top 20 genera, the abundances of *Arthrobacter*, *Gaiella*, *Microvirga*, *Blastococcus*, *Skermanella*, and *Candidatus Entotheonella* were significantly reduced in G compared with CM soil (*P* < 0.05), while *Nitrospira* and *Sphingobium* were increased significantly (*P* < 0.05, Table S2). Moreover, the relative abundances of these eight genera were similar to those of CM soil after three to five years of maize rotation (*P* > 0.05). No significant difference was observed in the relative abundance of other nitrifying bacteria, such as *Nitrolancea*, *Nitrosococcus* and *Nitrosomonas*, among all of the soil treatments (*P* > 0.05, Table S1).

**Figure 4:**
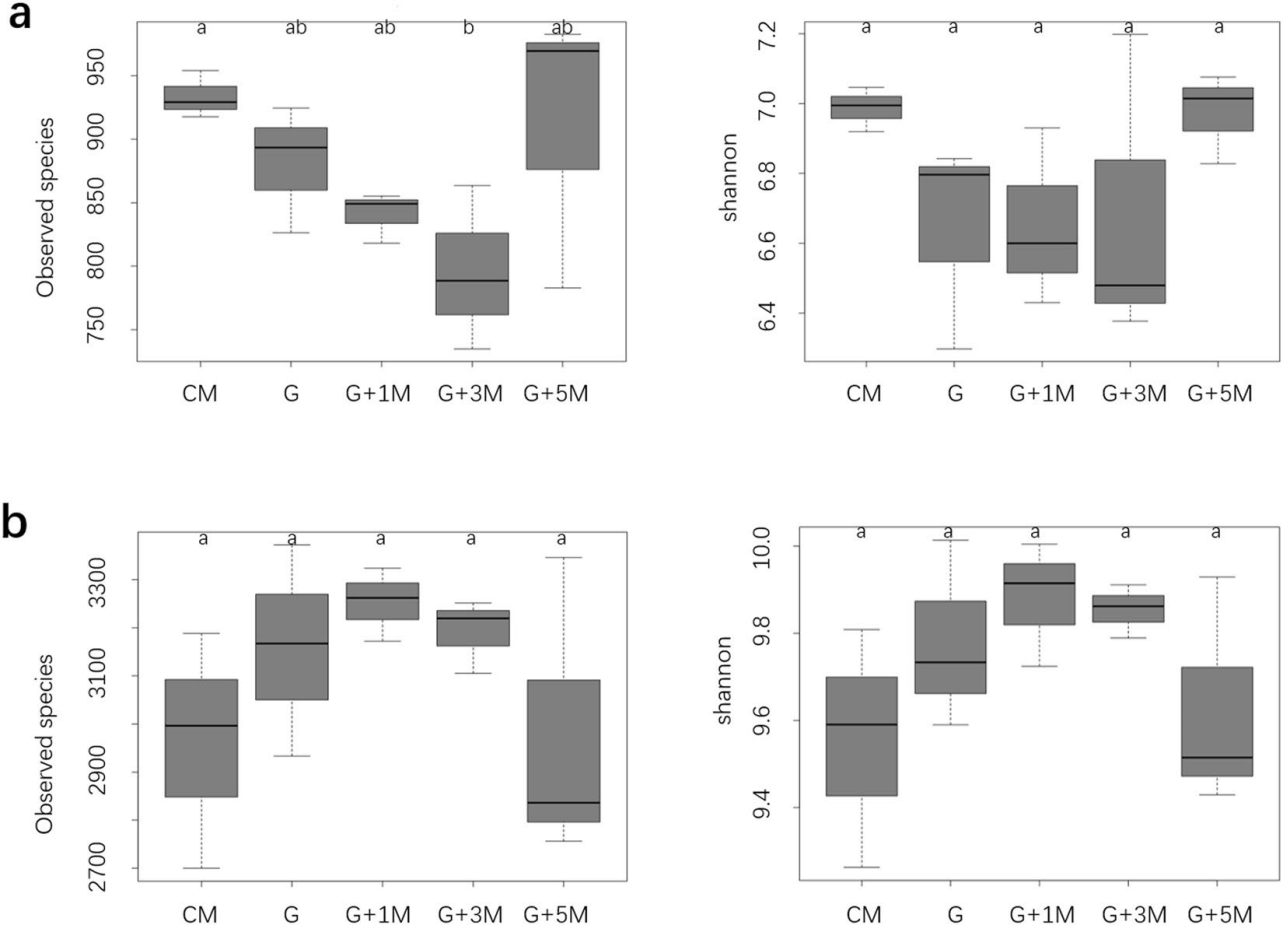
Fungal (**a**) and bacterial (**b**) diversity in the soil samples. The same letter above bars represents no significant difference (*P* < 0.05) according to Fisher’s least significant difference method.

Compared with CM soil, the values of the observed species index and Shannon diversity index of the fungal community were 6% and 5% lower in G soil, although no significant difference was observed (*P* > 0.05). Both values tended to increase to similar levels to those in CM after 5-year maize rotations (Fig. 4a). The values of the observed bacterial species index and Shannon diversity of the bacterial community were higher in G, G + 1 M and G + 3 M soils than in CM and G + 5 M soils (*P* > 0.05, Fig. 4b). Cluster analysis based on OTUs revealed similar fungal and bacterial communities in G and G + 1 M soils, while the microbial communities from G + 3 M, G + 5 M and CM soil grouped together (Fig. 3).

### Relative abundance of soil pathogenic fungi

T-RFLP analysis provided information for the fungal pathogen species in soil samples. Ten species of pathogenic fungi, such as *Alternaria panax*, *Botrytis cinerea*, *Cylindrocarpon destructans*, *Rhizoctonia solani* and some *Fusarium* spp., were identified by comparing them with the fungal T-RFLP patterns database (Table S3). The ratio of *A*. *panax* (TRF of 86 bp) in G and CM was similar but significantly lower than in G + 1 M (*P* < 0.05). The ratio of *Fusarium* spp. (TRF of 148 bp) in G + 5 M and CM was similar but significantly higher than other groups (*P* < 0.05). No differences in the ratio of *F*. *solani* (TRF of 130 bp), *C*. *destructans* (TRF of 132 bp), *B*. *cinerea* (TRF of 143 bp), and *R*. *solani* (TRF of 325 bp) were observed among the groups (Table 1).

**Table 1:** Ratio of identified terminal restriction fragments (ITS1F-HEX) of pathogenic fungal ITS regions.

Groups	*Alternaria panax* (86 bp)*	*Fusarium solani* (130 bp)*	*Cylindrocarpon destructans* (132 bp)*	*Botrytis cinerea* (143 bp)*	*Fusarium* spp. (148 bp)*	*Rhizoctonia solani* (325 bp)^#^
CM	1.04 ± 0.97b	7.07 ± 2.34a	0.00 ± 0.00a	6.38 ± 3.63a	15.83 ± 4.72a	1.84 ± 2.45a
G	0.95 ± 0.69b	4.41 ± 1.37a	0.84 ± 0.60a	14.97 ± 14.27a	3.42 ± 1.53b	2.29 ± 2.02a
G + 1 M	2.56 ± 0.76a	6.87 ± 6.26a	10.29 ± 13.23a	5.03 ± 1.06a	5.75 ± 3.73b	1.13 ± 0.24a
G + 3 M	1.28 ± 0.53ab	5.63 ± 2.04a	0.64 ± 0.40a	2.95 ± 2.91a	5.94 ± 3.62b	1.00 ± 1.73a
G + 5 M	1.16 ± 0.97ab	6.10 ± 2.44a	0.73 ± 0.64a	4.69 ± 4.26a	15.97 ± 6.21a	2.38 ± 2.21a

*indicates that TRF (ITS1F-HEX) was digested using *Hae*III, ^#^indicates that TRF (ITS1F-HEX) was digested by *Hinf*I. The means ± SD (n = 3) followed by the same letter are not significantly different in the same column, according to Fisher’s least significant difference method at α = 0.05. G =four-year cultivation of American ginseng, M = one-year cultivation of maize, CM = cultivated maize.

### Growth and morbidity of replanted American ginseng in the pot experiment

**Figure 5:**
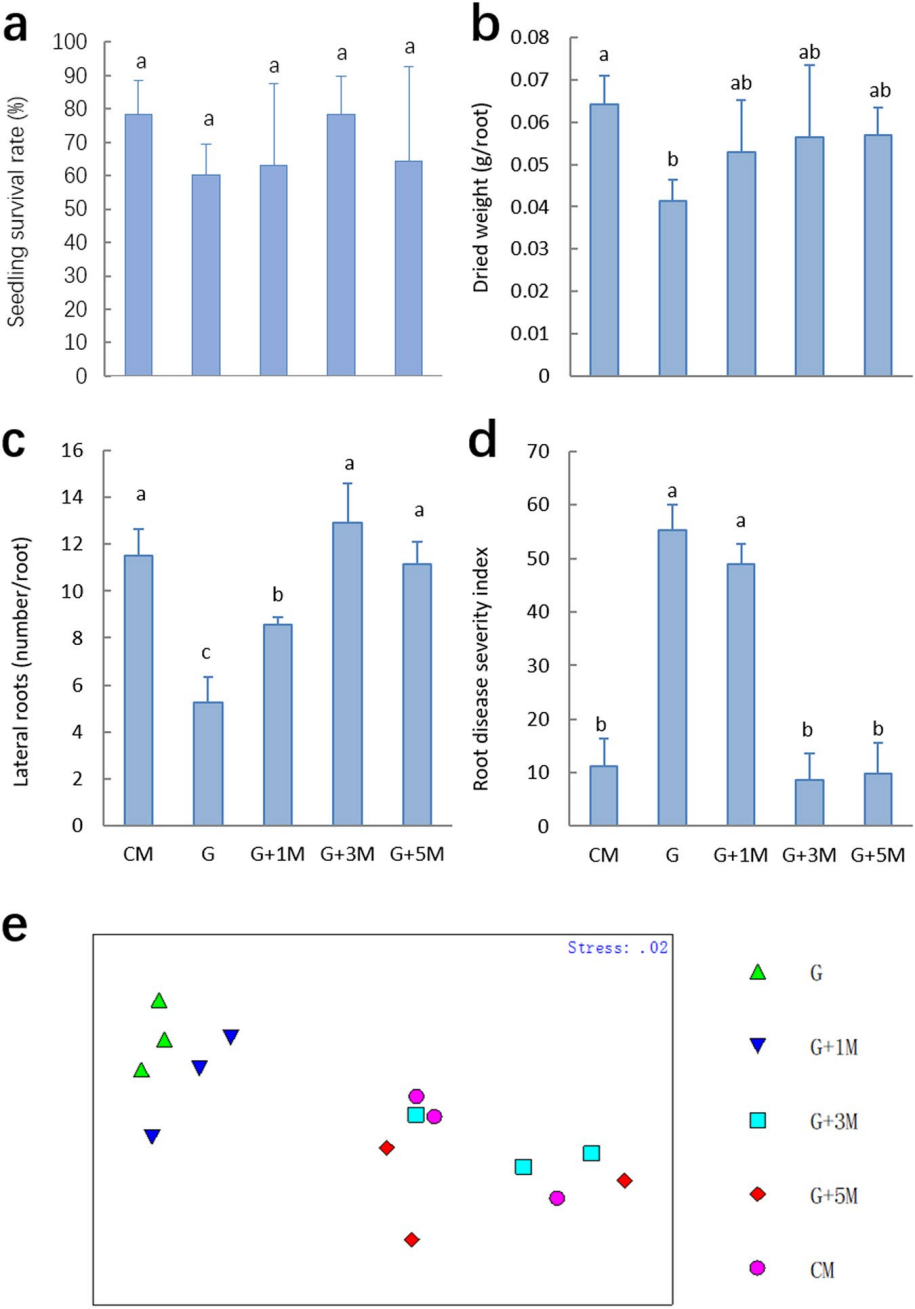
Seedling survival rate (**a**), root growth (**b**,**c**), and root disease severity (**d**) of American ginseng cultivated in different soils and non-metric multi-dimensional scaling (MDS) plot based on the data Log(X + 1) transformation and Bray-Curtis similarities (**e**). Error bars denote the standard deviation of the mean (n = 3), and data designated with the same letter are not significantly (*P* < 0.05) different, according to Fisher’s least significant difference method.

No significant differences were observed for the survival rates of seedlings among the treatments (Fig. 5a). The root-dried weight in G was significantly lower than that of CM (*P* < 0.05), while other treatments fell between these values (*P* > 0.05, Fig. 5b). The number of lateral roots was lowest in G, followed by G + 1 M, while the treatments of CM, G + 3 M, and G + 5 M had the highest number of lateral roots (*P > *0.05, Fig. 5c). The disease severity index of roots in G + 3 M, G + 5 M and CM was lowest, while it was dramatically higher in the G and G + 1 M soils (*P* < 0.05, Fig. 5d). All of the parameters were further analyzed with a non-metric multidimensional scaling (MDS) method by superimposing the group labels on the respective replicates. It became apparent that each of the three replicates from G/G + 1 M was quite different in plant growth conditions from G + 3 M/G + 5 M/CM (Fig. 5e). Our data showed that maize rotation showed an improvement in the growth of replanted American ginseng, as well as a reduction in the occurrence of root disease.

### Soil phenolic acids and microflora related to replanted ginseng growth and disease severity

Stepwise generation of multilinear regression models enabled an assessment of the relative importance of factors predicting ginseng growth and disease severity (Table S4). Among all the soil physicochemical and biological factors, root disease severity showed a positive correlation with soil *p*-hydroxybenzoic acid but a negative correlation with soil fungi diversity. The seedling survival rate, root yield, and lateral root number correlated positively with the soil fungal Shannon diversity. Lateral root numbers also correlated negatively with soil ferulic acid. However, neither the American ginseng growth index nor the root disease severity correlated with soil nutrients.

We further performed a Pearson’s correlation to evaluate the relationship between the replanted ginseng root disease severity and the dominant microbial genera. Among the top 20 abundant fungal and bacterial genera, Pearson’s correlation coefficients showed that the replanted ginseng root disease severity index was not correlated with the relative abundances of the top 20 fungal genera (*P* > 0.05, Table 2), but it was negatively correlated with the relative abundances of the bacterial genera *Arthrobacter*, *Gaiella*, *Microvirga*, *Skermanella*, *Roseiflexus*, and *Candidatus Entotheonella* and positively correlated with *Nitrospira* and *Sphingobium* (*P* < 0.05).

**Table 2:** Significant correlation coefficients between the root disease severity index of replanted American ginseng and the microbes (among 20 top abundant fungal and bacterial genera).

	Genus	Ginseng root disease severity index
Fungus	—	—
Bacteria	*Arthrobacter*	−0.582*
	*Gaiella*	−0.628*
	*Microvirga*	−0.565*
	*Skermanella*	−0.542*
	*Roseiflexus*	−0.585*
	*Candidatus Entotheonella*	−0.544*
	*Nitrospira*	0.604*
	*Sphingobium*	0.863*

“—” means no significant correlation between the ginseng root disease severity index and top 20 abundant fungal genera; *P < 0.05.

### Effects of soil chemical properties on microbial communities

The stepwise generation of the multilinear regression analysis results showed that all the soil fungal and bacterial observed species and the Shannon diversity indexes were positively related to pH (Table S5). The soil fungi observed species were also positively related to the vanillic acid content, while the Shannon diversity was negatively related to the *p*-hydroxybenzoic acid content. Moreover, the soil bacterial observed species index was positively related to the AP and TN content. No significant correlation was found between fungal and bacterial diversity.

## Discussion

It is believed that the imbalance of soil nutrients has contributed to replant failure^15^. Similarly, in this study, the G group (American ginseng cultivated soils for four years) were found to be rich in such soil nutrients as AN, AP, AK, available Mn, Cu, and Fe. We speculated that the enrichment of these soil available nutrients resulted from the higher chemical fertilizer inputs and the application of massive amounts of organic fertilizer (e.g., composted cattle manure and swine sludge, Table S6). Additionally, the enrichment of available Cu was mainly due to the application of fungicide (e.g., Bordeaux mixture). With maize rotation, the soil nutrients were generally reduced during the first three years. The lowest concentrations of most soil nutrients in G + 3 M was mainly observed because no fertilizer was applied to the maize crops until the third spring after harvesting of American ginseng (Table S6). In contrast to the other nutrients, an increasing trend of Ca content was found in the soils with the time of maize rotation, possibly because maize symbiotic or rhizospheric microorganisms can increase the avaliablity of Ca^29^. Regression analysis suggested that there was no correlation between all soil nutrients and replanted American ginseng growth or root disease severity, but the decreasing levels of some nutrients, such as Mn, B, TN and SOM, may be responsible for the favorable growth condition of replant ginseng in G + 3 M soil. However, cluster analysis showed that the soil nutrients of G and G + 1 M were similar, while G + 3 M, G + 5 M and CM were similar, possibly because the crop rotation balanced the nutrients in the soil^30^.

Higher contents of phenolic acids, such as *p*-hydroxybenzoic acid, syringic acid, ferulic acid, salicylic acid and *p*-coumaric acid, were detected in G soil, and most of them decreased sharply after the 1-year maize rotation, excluding *p*-hydroxybenzoic acid, which decreased significantly after 3 years. Similarly, Du *et al*. reported that rotation with maize reduced the concentrations of ferulic acid, *p*-hydroxybenzoic acid, and *p*-coumaric acid in soil cultivated with *Rehmannia glutinosa*^31^. Our results showed that phenolic acids were different between G and CM treatments because of the variation of crops. Most phenolic acids in G soil decreased after the 1-year maize rotation and established a new balance. For vanillic acid and vanillin, no significant difference was found among all the treatments, suggesting that the effects of maize and American ginseng on the two individual phenolic acids were similar. Although the contents of most phenolic acids showed similar levels among soils with maize cropping (G + 1 M, G + 3 M, G + 5 M and CM), the growth of replanting ginseng cultivated in G + 1 M soil was still not as good as other soils, suggesting that soil phenolic acids were not restrictive for replanting American ginseng.

Soil microorganisms play an important role in the soil environment. Our sequencing results showed that ginseng potential pathogenic fungal genera, such as *Fusarium*, *Alternaria*, and *Cylindrocarpon*, did not change significantly among all of the soil groups. Moreover, the relative abundances of the root pathogens *Fusarium solani*, *Cylindrocarpon destructans* and *Rhizoctonia solani* showed a similar trend based on T-RFLP analysis. However, some soil bacteria, such as the *Arthrobacter*, *Gaiella*, *Microvirga*, *Skermanella*, *Roseiflexus*, and *Candidatus Entotheonella* genera, were increased by maize rotation. Among these bacteria, previous studies have shown that some genera are beneficial. Li *et al*. (2017) reported that some species of *Arthrobacter* can degrade allelochemicals^32^. Ardley *et al*. (2012) reported that *Microvirga* can fix nitrogen^33^, and Zhao *et al*. (2017) reported that several *Microvirga* isolates show strong resistance to plant pathogens^34^. Our correlation regression analysis further demonstrated that the disease severity of replanted ginseng root was significantly related to the relative abundance of these beneficial bacteria rather than pathogenic fungi. Previous studies reported that even though virulent pathogens were present in healthy soils, the occurrence of soil-borne plant disease was low because of the interactions and restrictions existing among soil phytopathogenic, beneficial, and commensal microbes^35,36^. Zhao *et al*. (2017) found that maize cultivation could enhance the soil microenvironment for the growth of beneficial microorganisms rather than *F*. *oxysporum*, to improve soil functions and overcome the replant problem of *P*. *notoginseng*^37^, consistent with our results.

Our results implied that the root disease of American ginseng was related to soil fungal diversity, which was likely affected by soil physicochemical properties. In concert with our findings, Dong *et al*. (2016) reported that the death rate of notoginseng and the soil fungal diversity were significantly negatively correlated^13^. Xiong *et al*. (2017) reported that the suppressive soil of vanilla *Fusarium* wilt disease harbored a higher fungal diversity but a lower bacterial diversity than the conducive soil, and the microbial diversity indices were correlated with the soil pH^27^. Birgander *et al*. (2014) reported that soil fertility affected fungal communities along a grassland gradient, and pH was one of the main factors controlling the microbial communities^38^. Wu *et al*. (2015) reported that a phenolic acid mixture could promote mycelial growth, sporulation, and toxin production of pathogenic *Fusarium oxysporum* while inhibiting the growth of beneficial *Pseudomonas* sp., suggesting that phenolic acids have varying effects on soil fungi^39^.

Our pot experiment results indicate that maize rotation over 3 years may improve growth of replanted American ginseng. American ginseng growth in the soil with three or five years of maize rotations was not significantly different than that in CM soil. The cluster analysis of soil nutrients, autotoxic phenolic acids, and fungal and bacterial communities showed that G + 3 M, G + 5 M, and CM soil were mostly grouped together. Plants can shape the specific microbial community structure in the soil environment^22^. Specifically, the initial soil microbial communities are shaped by the preceding crops, but they become more specific to subsequent crops in a rotation system^40^. The establishment of the soil microbiota for a plant is a dynamic process. It takes time for the soil microbial community to shift from one host to another. Our findings suggested that three or more years are needed for the microbial community in ginseng cultivated soil to become similar to maize soil. Combining the change pattern in soil physical and chemical properties and the microbial communities, we suggest that to eliminate the inhibitory factors of G soil, the suitable time for replanting ginseng by maize rotation is likely more than 3 years. Our findings provide useful information for the establishment of the American ginseng-maize-American ginseng rotation system.

## Conclusions

Our study demonstrates that maize rotation can change the physicochemical and biological properties of American ginseng cultivated soil, including soil nutrients, phenolic acid contents, and soil fungal and bacterial communities. The improvement of soil by maize rotation was mainly related to the alteration of the soil microbial community structure, such as an increased relative abundance of beneficial bacteria *Arthrobacter*, and the degradation of phenolic acids, such as *p*-hydroxybenzoic and ferulic acids, but not to the soil nutrients and abundance of pathogenic fungi. The maize rotation substantially alleviated the replant disorders of ginseng, and at least three years of maize rotation provided an effective break period before ginseng replanting. Our research findings provide new insights to sustain ginseng productivities that will benefit farmers in ginseng production regions. Future studies are needed to explore the interactions among soil nutrients, phenolic acids, and soil microbes to improve our understanding of the complicated ginseng replant problems.

## Materials and Methods

### Field investigation and soil sampling

Field investigation was conducted in loam soil (mollic gleysols) in Huairou District, Beijing (116°37′E, 40°44′N). Depending on the years with maize rotations after ginseng cultivation, we selected the following groups for soil samplings: continuous maize (CM), ginseng (G), ginseng plus 1-year maize (G + 1 M), ginseng plus 3-year maize (G + 3 M), and ginseng plus 5-year maize (G + 5 M). Originally, these fields were used for annual spring maize with a production of 6.0–6.8 t ha^−1^ and were kept fallow during winter. Afterwards, the fields were grown with American ginseng for four years continuously, and then the fields were again used for maize crops (Table S6). Each group included three fields (called replicates in this study), and the area of the fields ranged from 0.4 to 2 ha. All the fields were <50 km apart. For each crop, agronomic practices, such as irrigation, fertilization, and weeding, were the same in all the fields. In the autumn of 2011, while ginseng root or maize was manually harvested, surface soil of 0–20 cm was sampled. Within each plot, multiple sampling locations were selected, and approximately 30 kg of soil was collected. The soil samples were homogenized and sieved with a 2-mm mesh sieve to remove large pieces of debris. One part of each sample was stored at −80 °C for subsequent DNA extraction, another portion was air-dried for chemical analysis, and the rest was used in the pot experiment.

### Analysis of the soil chemical properties

Approximately 100 g of soil samples was sieved through a 20-mesh sieve (0.84-mm opening diameter) to determine the soil-available nutrients and through a 100-mesh sieve (0.15-mm opening diameter) to determine the total nutrients and soil organic matter (SOM). The total nitrogen (TN) and total phosphorus (TP) were analyzed following the procedure of Fawcett *et al*.^41^ and the Chinese Ministry of Environmental Protection^42^. Available nitrogen (AN), available phosphorus (AP), and available potassium (AK) were determined by chemical analytical methods, as described by Liu *et al*.^43^, Murphy *et al*.^44^, and Hanway *et al*.^45^. Soil organic matter (SOM) was assayed according to the Walkley-Black method^46^. Soil pH was determined at a soil:water ratio of 1:2 using a combination glass electrode. The exchangeable calcium (Ca), magnesium (Mg), and available sulfur (S), boron (B), iron (Fe), manganese (Mn), copper (Cu), and zinc (Zn) were extracted and determined using an inductively coupled plasma optical emission spectrometer (ICAP6300, Thermo Fisher Scientific, US) according to Chinese Agricultural Standards^47^. Soil moisture content was determined after oven-drying at 105 °C for 6 h.

### Analysis of the phenolic acids in soil

Seven phenolic acids, including *p*-hydroxybenzoic acid, vanillic acid, syringic acid, vanillin, *p*-coumaric acid, ferulic acid, and salicylic acid, were extracted from the soil after passing through a 0.3-mm mesh and assessed by high-performance liquid chromatography (HPLC, Waters, TM 2695 pump/2996 UV-DAD detector/7752 injector, Milford, MA), following the method of Zhang *et al*.^48^. These compounds were separated by a YMC-Pack ODS-A column (250 mm × 4.6 mm, 5 µm) and identified and quantified by comparison to their corresponding standards (obtained from Alfa Aesar Co., UK) (Table S7).

### Microbial community analysis

The microbial community was analyzed following a procedure of DNA extraction, PCR amplification and Illumina MiSeq sequencing. DNA was extracted from 0.4 g of soil sample using the MOBIO PowerSoil® DNA Isolation Kit (Mobio Laboratories Inc., Carlsbad, CA, USA). The fungal universal ITS1 region was amplified with the primers ITS1-F (5′-CTTGGTCATTTAGAGGAAGTAA-3′) and ITS2 (5′-TGCGTTCTTCATCGATGC-3′)^49^. The bacterial universal V3-V4 region of the 16S rRNA gene was amplified with the primers 338F (5′-ACTCCTACGGGAGGCAGCAG-3′) and 806R (5′ -GGACTACHVGGGTWTCTAAT-3′)^50^. PCR volumes were 50 μL, containing 30 ng template DNA, 0.3 μL DNA polymerase (Takara Biotechnology, Dalian, CO., LTD), 2 μL of 10 μM of each primer, 5 μL 10× Pyrobest Buffer, and 4 μL 2.50 mM dNTP. PCR amplification was carried out using the following protocol: 95 °C for 5 min, followed by 35 cycles (for fungal ITS1 region) or 28 cycles (for bacterial V3-V4 region) at 95 °C for 45 s, annealing at 55 °C for 50 s, 72 °C for 45 s and a final extension at 72 °C for 10 min. Three PCR products per sample were pooled, purified, and quantified by real-time PCR. Parallel-tagged sequencing was performed on an Illumina MiSeq platform (Allwegene, Beijing, China) according to standard protocols^51,52^. Specifically, split reads were merged using FLASH V1.2.11 and sorted into each sample by the unique barcodes using QIIME (V1.7.0). The raw data were first screened, and sequences were removed by considering whether their quality scores <20 contained ambiguous bases or did not exactly match the primer sequences and barcode tags. Raw tags with <200 bp were removed using Mothur (V1.37.0). Chimeras were removed with USEARCH (V8.1.1861) against the Gold and UNITE reference database. The high-quality sequences were clustered into operational taxonomic units (OTUs) at a threshold of 97% similarity using the UPARSE pipeline. Singletons that occur only once in the entire data set were removed from subsequent analyses to reduce overprediction of rare OTUs. The representative OTU sequences were aligned and annotated using the Ribosomal Database Project (RDP) classifier (V14). Alpha diversities such as the observed species and Shannon diversity index were analyzed using QIIME (V1.7.0).

### Fungal pathogens analysis

Fungal pathogen analysis was based on PCR followed by restriction digestion and T-RFLP analysis. Soil sample DNA was amplified with universal PCR primers of ITS1F labeled with HEX (5-hexachloro-fluorescein) and ITS4 labeled with FAM (5-FAM [5-Carboxyfluorescein]) according to previous methods^53,54^. PCR products were digested with *Hinf*I or *Hae*III according to Dickie *et al*.^55^. Digested PCR products were electrophoresed on an automated sequencer (ABI PRISM1 3730 × l Genetic Analyzer) and then analyzed using Peak scanner software 1.0 (Applera Corporation®, USA). Ten species of pathogenic fungi were identified through comparison of the T-RFLP patterns between soil samples and a database of pure hypha (Table S3). The ratios of most pathogenic fungus were calculated by the peak area of TRF produced by *Hae*III digestion and HEX-labeled primer (ITS1F), while the ratio of *R*. *solani* (325 bp) was calculated from the results with *Hinf*I and HEX (Table S3).

### Pot experiment for ginseng growth

A pot experiment was conducted with the soils from the fields of CM, G, G + 1 M, G + 3 M, and G + 5 M. The soils from the triplicate field plots of each group were mixed thoroughly, and approximately 25 kg of the soil was added to one clay pot (50 cm in height, 40 cm in diameter). There were three replicates for each treatment. All the pots were placed randomly without fertilizer and pesticide amendments. Fifty American ginseng seeds were sown in each pot. The pot experiment was conducted with regular irrigation and artificial shade with a double-sunshade cloth to reduce solar radiation to an optimal 15% to 25% of full sunlight. The weeds were removed manually once a week. The seedling emergence rate was examined 60 days after sowing. All plants were harvested 5 months after sowing. The relative survival rate of seedlings, dry weight of individual roots, root disease severity index, and number of lateral roots were measured.

### Data analysis

All data were analyzed using analysis of variance (ANOVA) with SPSS19.0 (SPSS Inc., Chicago, USA). Differences between groups were compared by Fisher’s least significant difference test. One-way ANOVA was performed with a *P* value < 0.05. Soil nutrient and phenolic acid data were further analyzed by cluster analysis using the between-group linkage method and squared Euclidean distance measure. Clustering analyses of soil microbes were calculated with unweighted UniFrac distance matrices, with the arithmetic mean (UPGMA) clustering procedure to establish the dendrogram using R software (Version 2.15.3) based on the OTU information from each sample. Pearson’s correlation coefficient was used to evaluate the relationships between the replanted ginseng root disease severity and top 20 fungal or bacterial genera. The root disease severity observed in pot experiment was assessed by the presence of surface lesions, which were quantified from 0 to 4, where 0 = no lesions, 1 ≤ 10%, 2 = 10–33%; 3 = 33–67% and 4 ≥ 67% of the total surface area with rot.

$${\rm{R}}{\rm{o}}{\rm{o}}{\rm{t}}\,{\rm{d}}{\rm{i}}{\rm{s}}{\rm{e}}{\rm{a}}{\rm{s}}{\rm{e}}\,{\rm{s}}{\rm{e}}{\rm{v}}{\rm{e}}{\rm{r}}{\rm{i}}{\rm{t}}{\rm{y}}\,{\rm{i}}{\rm{n}}{\rm{d}}{\rm{e}}{\rm{x}}=\frac{{\rm{\Sigma }}({\rm{V}}{\rm{a}}{\rm{l}}{\rm{u}}{\rm{e}}\,{\rm{o}}{\rm{f}}\,{\rm{d}}{\rm{i}}{\rm{s}}{\rm{e}}{\rm{a}}{\rm{s}}{\rm{e}}\,{\rm{s}}{\rm{c}}{\rm{a}}{\rm{l}}{\rm{e}}\times {\rm{n}}{\rm{u}}{\rm{m}}{\rm{b}}{\rm{e}}{\rm{r}}\,{\rm{o}}{\rm{f}}\,{\rm{r}}{\rm{o}}{\rm{o}}{\rm{t}}{\rm{s}})}{{\rm{H}}{\rm{i}}{\rm{g}}{\rm{h}}{\rm{e}}{\rm{s}}{\rm{t}}\,{\rm{v}}{\rm{a}}{\rm{l}}{\rm{u}}{\rm{e}}\,{\rm{o}}{\rm{f}}\,{\rm{d}}{\rm{i}}{\rm{s}}{\rm{e}}{\rm{a}}{\rm{s}}{\rm{e}}\,{\rm{s}}{\rm{c}}{\rm{a}}{\rm{l}}{\rm{e}}\times {\rm{t}}{\rm{o}}{\rm{t}}{\rm{a}}{\rm{l}}\,{\rm{n}}{\rm{u}}{\rm{m}}{\rm{b}}{\rm{e}}{\rm{r}}\,{\rm{o}}{\rm{f}}\,{\rm{r}}{\rm{o}}{\rm{o}}{\rm{t}}{\rm{s}}}\times 100$$

Similarity analysis of the growth index of American ginseng among groups was analyzed using non-metric multi-dimensional scaling (MDS) based on data Log(X + 1) transformation. Following 100 random restarts, the ordinations in our analyses were computed with the best final configuration, based on the number of times that a minimum stress was obtained over the course of the random restarts^56^. Multilinear regression analysis was conducted using SPSS19.0, and all data were transformed in scientific notation.

## Supplementary information

Supplementary table

## Data Availability

Raw data are available upon request.
