# Supplementary material for: Effects of maize rotation on the physicochemical properties and microbial communities of American ginseng cultivated soil
Source: Sci Rep. 2019 Jun 13;9:8615. doi: 10.1038/s41598-019-44530-7 (PMC6565631; doi:10.1038/s41598-019-44530-7)
Supplement: Supplementary file 1 — Supplementary table [file 41598_2019_44530_MOESM1_ESM.docx]

**Effects of maize rotation on the** **physicochemical properties and microbial communities of American ginseng cultivated soil**

Xiao-Lin Jiao#^1^, Xue-Song Zhang#^1^, Xiao-Hong Lu^1,3^, Ruijun Qin^2^, Yan-Meng Bi^1^ & Wei-Wei Gao*^1^

1 Institute of Medicinal Plant Development, Chinese Academy of Medical Sciences and Peking Union Medical College, Beijing 100193, China.

2 Oregon State University-Hermiston Agricultural Research and Extension Center, Hermiston, OR 97838, USA.

3 Institute of Plant Protection, Chinese Academy of Agricultural Sciences, Beijing 100193, China.

#These authors contributed equally to this work.

*Corresponding author

E-mail: [wwgao411@sina.com](mailto:wwgao411@sina.com)

Tel: +86-10-57833423, Fax: +86-10-57833020

**Supplementary table**

**Table S1. Relative abundances of microbial functional genera in different soil categories identified by Illumina MiSeq sequencing**

|  |  | CM | G | G+1M | G+3M | G+5M |
| --- | --- | --- | --- | --- | --- | --- |
| Fungi | *Alternaria* | 0.36±0.11a | 0.09±0.03a | 0.29±0.28a | 0.28±0.2a | 0.23±0.03a |
|  | *Cylindrocarpon* | 0.08±0.07a | 0.02±0.01a | 0.06±0.02a | 0.16±0.14a | 0.13±0.05a |
|  | *Penicillium* | 0.23±0.2a | 0.26±0.08a | 0.09±0.03a | 0.13±0.07a | 0.23±0.1a |
|  | *Trichoderma* | 0.23±0.27a | 0.03±0.01a | 0.05±0.03a | 0.16±0.25a | 0.07±0.08a |
|  | *Glomus* | 0.06±0.04a | 0.04±0.02a | 0.19±0.24a | 0.15±0.14a | 0.41±0.38a |
| Bacteria | *Nitrolancea* | 0.04±0.03a | 0.04±0.04a | 0.03±0.01a | 0.04±0.04a | 0.05±0.04a |
|  | *Nitrosococcus* | 0.01±0.006a | 0.004±0.002a | 0.008±0.008a | 0.02±0.012a | 0.01±0.002a |
|  | *Nitrosomonas* | 0.04±0.03a | 0.02±0.02a | 0.01±0.006a | 0.02±0.01a | 0.02±0.01a |

**Table S2. Relative abundances of the 20 top abundant bacterial genera in soil samples**

|  |  | CM | G | G+1M | G+3M | G+5M |
| --- | --- | --- | --- | --- | --- | --- |
| 1 | ***Arthrobacter*** | 2.79±1.17a | 1.16±0.25b | 1.87±0.56ab | 1.97±0.33ab | 2.51±0.59a |
| 2 | *Sphingomonas* | 1.91±0.37a | 2.4±1.30a | 1.98±0.45a | 1.33±0.06a | 2.42±0.57a |
| 3 | ***Gaiella*** | 2.19±0.47a | 0.65±0.27c | 1.05±0.47bc | 1.26±0.37bc | 1.46±0.41ab |
| 4 | *Bacillus* | 1.30±0.91a | 0.87±0.55a | 0.88±0.32a | 0.80±0.36a | 1.17±0.70a |
| 5 | ***Microvirga*** | 1.45±0.22a | 0.51±0.14b | 0.76±0.27ab | 0.83±0.13ab | 1.21±0.7ab |
| 6 | ***Blastococcus*** | 1.69±1.00a | 0.53±0.16b | 0.71±0.33ab | 0.64±0.16b | 1.03±0.41ab |
| 7 | *Acidibacter* | 0.75±0.28a | 0.73±0.07a | 0.86±0.15a | 1.08±0.27a | 1.10±0.32a |
| 8 | ***Skermanella*** | 1.53±0.17a | 0.49±0.15b | 0.62±0.07b | 0.73±0.25b | 1.14±0.68ab |
| 9 | *Blastocatella* | 0.72±0.34a | 0.84±0.42a | 0.79±0.13a | 0.99±0.19a | 1.14±0.62a |
| 10 | *Haliangium* | 0.62±0.16b | 0.72±0.05ab | 0.93±0.04a | 0.85±0.12a | 0.78±0.13ab |
| 11 | *Roseiflexus* | 1.09±0.73a | 0.38±0.17a | 0.49±0.16a | 0.75±0.18a | 0.95±0.31a |
| 12 | ***Nitrospira*** | 0.38±0.19b | 0.96±0.31a | 0.72±0.20ab | 0.68±0.13ab | 0.56±0.10b |
| 13 | ***Candidatus***  ***Entotheonella*** | 1.07±0.22a | 0.34±0.09b | 0.51±0.2b | 0.57±0.22b | 0.68±0.32ab |
| 14 | *Bryobacter* | 0.46±0.02c | 0.54±0.08bc | 0.78±0.08a | 0.60±0.10bc | 0.65±0.13ab |
| 15 | *Chryseolinea* | 0.44±0.30a | 0.53±0.19a | 0.72±0.17a | 0.70±0.13a | 0.46±0.13a |
| 16 | *Steroidobacter* | 0.47±0.14a | 0.48±0.10a | 0.71±0.26a | 0.62±0.16a | 0.54±0.06a |
| 17 | *Lysobacter* | 0.65±0.48a | 0.20±0.07a | 0.60±0.16a | 0.57±0.06a | 0.63±0.30a |
| 18 | *Rubrobacter* | 0.89±0.77a | 0.36±0.20a | 0.40±0.17a | 0.30±0.06a | 0.61±0.48a |
| 19 | *Altererythrobacter* | 0.61±0.20a | 0.48±0.12ab | 0.33±0.13b | 0.41±0.12ab | 0.58±0.09ab |
| 20 | ***Sphingobium*** | 0.06±0.04c | 1.52±0.10a | 0.57±0.24b | 0.05±0.02c | 0.12±0.07c |

**Table S3. Species, resources, and lengths (bp) of terminal restriction fragments of the identified pathogenic fungus of the ITS (ITS1F/ITS4) region using *Hae*III and *Hinf*I digestion**

| No. | Species | Sources | *Hae*III terminal fragments (bp) | | *Hinf*I terminal fragments (bp) | |
| --- | --- | --- | --- | --- | --- | --- |
|  |  |  | ITS4 (5'FAM) | ITS1F (5'HEX)* | ITS4 (5'FAM) | ITS1F (5'HEX)* |
| 1 | *Alternaria panax* | Leaf of *P. ginseng* | 135 | **86** | 161 | 316 |
| 2 | *Botrytis cinerea* | Root of *P. ginseng* | 429 | **143** | 174 | 299 |
| 3 | *Cylindrocarpon destructans* | Root of *P. quinquefolium* | 87 | **132** | 276 | 287 |
| 4 | *Fusarium avenaceum* | Soil that *P. quinquefolium* grown | 85 | **148** | 267 | 299 |
| 5 | *F. oxysporum* | Root of *P. quinquefolium* | 85 | **148** | 177 | 299 |
| 6 | *F. equiseti* | Root of *Achyranthes bidentata* | 85 | **149** | 175 | 300 |
| 7 | *F. solani* | Root of *P. quinquefolium* | 86 | **130** | 44 | 301 |
| 8 | *F. verticillioide* | Soil that *P. quinquefolium* grown | 86 | **148** | 279 | 299 |
| 9 | *F. proliferatum* | Root of *P. quinquefolium* | 86 | **148** | 279 | 299 |
| 10 | *Rhizoctonia solani* | Stem of *P. quinquefolium* | 118 | 99 | 278 | **325** |

*The numbers in bold font were used for identification of fungal strains in soil samples.

**Table S4. Models obtained by stepwise linear regression of soil chemical properties and soil microbial properties for disease suppression, seedling survival, root biomass, and lateral root numbers**

| Dependent variable | Independent variable | Regression  coefficient^*^ | *P* | *R*^2^ |
| --- | --- | --- | --- | --- |
| Root disease severity | *p*-Hydroxybenzoic acid | 20.281 | 0.000 | 0.938 |
|  | Fungi observed species | -2.487 | 0.004 |  |
| Seedling survival rate | Shannon index for fungi | 1.016 | 0.000 | 0.945 |
| Root biomass | Shannon index for fungi | 0.316 | 0.000 | 0.965 |
| Lateral roots number | Shannon index for fungi | 1.704 | 0.000 | 0.978 |
|  | Ferulic acid | -2.243 | 0.000 |  |

**Table S5. Models obtained by stepwise linear regression of soil chemical properties for soil microbial diversity**

| Dependent variable | Independent variable | Regression  coefficient^*^ | *P* | *R*^2^ |
| --- | --- | --- | --- | --- |
| Fungi observed species | pH | 1.134 | 0.000 | 0.995 |
|  | Vanillic acid | 0.686 | 0.045 |  |
| Shannon diversity for fungi | pH | 0.984 | 0.000 | 0.999 |
|  | *p*-Hydroxybenzoic acid | -0.163 | 0.036 |  |
| Bacterial observed species | pH | 0.044 | 0.000 | 0.998 |
|  | AP | 0.024 | 0.004 |  |
|  | TN | 0.270 | 0.020 |  |
| Shannon diversity for bacteria | pH | 1.335 | 0.000 | 1.000 |

**Table S6. Crop patterns of the collected field soil samples**

| Groups | 2002 | 2003 | 2004 | 2005 | 2006 | 2007 | 2008 | 2009 | 2010 | 2011* |
| --- | --- | --- | --- | --- | --- | --- | --- | --- | --- | --- |
| CM | M | M | M | M | M | M | M | M | M | M |
| G | - | - | - | M | M | M | G | G | G | G |
| G+1M | - | - | M | M | M | G | G | G | G | M^a^ |
| G+3M | M | M | M | G | G | G | G | M^a^ | M^a^ | M^b^ |
| G+5M | M | G | G | G | G | M^a^ | M^a^ | M^b^ | M | M |

M: maize; G: American ginseng. - indicates the cultivated plant is not clear; *indicates the year of soil sample collected. For ginseng cultivation, a combination of composted cattle manure and swine sludge (2:1 *w/w*) was applied at a rate of 45–60 t/ha in the early spring before ginseng seeding, and 7.5 t/ha organic fertilizer consisting of a mixture of composted cattle manure and swine sludge (2:1 *w/w*) was applied in late October of each growing season. For maize cultivation, fertilizers including 345 kg/ha N(CO(NH_2_)_2_), 137 kg/ha P(P_2_O_5_) and 180 kg/ha K(K_2_SO_4_) were applied each year, except for the following: a): not fertilized, b): a compound fertilizer (150–375 kg/ha (NH_4_)^2^HPO_4_ and 150–300 kg/ha CO(NH_2_)_2_) was applied.

**Table S7. Standard curve and recovery of each phenolic acid detected using HPLC**

| **Phenolic acid** | **Equations of standard curve^＊^** | ***R²*** | **Linear ranges/μg** | **Recovery (%)**  **Mean ± SD, n = 6** |
| --- | --- | --- | --- | --- |
| ***p*-Hydroxybenzoic acid** | *y* = 1313000.67*x* − 2599.36 | 0.9999 | 0.0083–0.4144 | 99.01 ±3.22 |
| **Vanillic acid** | *y* = 1444053.84*x* − 3094.07 | 0.9998 | 0.0073–0.3670 | 92.34 ± 3.88 |
| **Syringic acid** | *y* = 3141900.70*x* − 4703.47 | 0.9999 | 0.0068–0.3383 | 97.37 ± 2.69 |
| **Vanillin** | *y* = 4874759.87*x* − 10883.06 | 0.9999 | 0.0101–0.5064 | 95.60 ± 3.84 |
| ***p*-Coumaric acid** | *y* = 6401167.73*x* − 24559.44 | 0.9999 | 0.0148–1.4800 | 94.68 ±2.03 |
| **Ferulic acid** | *y* = 2926792.50*x* − 7473.48 | 0.9999 | 0.0146–0.3670 | 92.46 ± 3.72 |
| **Salicylic acid** | *y* = 434988.58*x* − 1931.89 | 0.9995 | 0.00398–0.3976 | 89.48 ± 4.13 |

^＊^*y* is the peak area in HPLC, and *x* is the concentration of the corresponding compound.
